# Multicentre Evaluation of AI-Assisted Caries Detection on Panoramic Radiographs Among Early-Career Dentists

**DOI:** 10.1016/j.identj.2026.109812

**Published:** 2026-09-08

**Authors:** Yujia Wu, Xiaowei Hou, Peng Ding, Zineng Xu, Hailong Bai, Yan Liu, Lili Chen, Mingming Xu, Xuliang Deng

**Affiliations:** a Department of Geriatric Dentistry, Peking University School and Hospital of Stomatology, National Center for Stomatology, National Clinical Research Center for Oral Diseases, National Engineering Research Center of Oral Biomaterials and Digital Medical Devices, Beijing, China; b Department of Stomatology, Third Hospital of Hebei Medical University, Shijiazhuang, China; c DeepCare Inc., Beijing, China; d Clinical Trial Organization, Peking University School and Hospital of Stomatology, National Center for Stomatology, National Clinical Research Center for Oral Diseases, Beijing, China; e Hospital of Stomatology, Guanghua School of Stomatology, Sun Yat-sen University, Guangzhou, China; f Guangdong Provincial Key Laboratory of Stomatology, Guangzhou, China; g National Medical Products Administration Key Laboratory of Dental Materials, Beijing, China

**Keywords:** Artificial intelligence, Panoramic radiography, Dental caries, Clinical decision support

## Abstract

**Introduction and aims:**

Artificial intelligence – assisted diagnostic tools are increasingly introduced into dental practice, yet their impact on clinician performance in routine panoramic radiograph interpretation remains incompletely defined. This multicentre reader study evaluated whether AI assistance improves diagnostic accuracy, efficiency, and inter-reader consistency in caries detection, particularly among early-career dentists.

**Methods:**

Twelve early-career dentists (≤3 years’ experience) and three senior dentists (>10 years’ experience) independently interpreted 402 anonymized panoramic radiographs under four conditions: unassisted reading, AI-assisted reading, AI-only analysis, and expert reference. Diagnostic performance was compared with a standardized expert-derived reference standard established by three experienced dentists using pixel-level annotations. Sensitivity, specificity, area under the receiver operating characteristic curve, interpretation time, and inter-reader agreement were analysed.

**Results:**

AI assistance increased tooth-level sensitivity (82.4% vs 67.4%, *P* < .001) without compromising specificity (97.4% vs 97.2%), and reduced mean interpretation time (50.37 vs 65.12 seconds, *P* = .003). Case-level sensitivity improved from 84.7% to 93.3% (*P* < .001). Inter-reader agreement increased from *κ* = 0.61 to 0.73. The standalone AI system achieved a sensitivity of 79.2% (95% CI, 76.0-82.4) and specificity of 98.4% (95% CI, 76.0-82.4), and AUC of 0.938 (95% CI, 0.934–0.941).

**Conclusion:**

AI assistance improved diagnostic sensitivity, efficiency, and consistency among early-career dentists interpreting panoramic radiographs for caries detection, without increasing false-positive rates.

**Clinical relevance:**

AI-supported interpretation may help reduce experience-related variability in panoramic radiograph assessment and improve diagnostic efficiency in routine dental practice, particularly in settings with limited access to senior supervision.

## Introduction

Dental caries is among the most common chronic conditions worldwide, with the 2022 WHO Global Oral Health Report estimating that 2.5 billion people live with untreated decay.^1^ In China, the situation is especially pronounced: recent epidemiological studies indicate that over 88% of adults are affected, with prevalence rising to nearly 98% among older adults.^2^ Although early detection plays a critical role in enabling minimally invasive treatment, clinicians face several practical challenges. The high volume of images requiring interpretation renders sole reliance on manual reading time-consuming and inefficient. Inter-reader variability and the risk of missed lesions are further exacerbated in busy clinical settings. Panoramic radiographs have gained traction as a practical screening modality because they provide a comprehensive view of the dentition with lower radiation exposure than cone-beam computed tomography, while remaining patient-friendly.^3^ However, the broad field of view and overlapping anatomical structures can obscure subtle lesions, resulting in variable diagnostic performance between early-career and experienced dentists.

Panoramic radiographs acquired for routine clinical purposes may incidentally reveal carious lesions, although diagnostic performance varies substantially between readers.^4^ However, substantial diagnostic disparities remain: one study found that general dentists miss up to 61% of carious lesions relative to specialists.^5^ These discrepancies stem from variations in radiographic training, diagnostic thresholds, and intrinsic limitations of panoramic imaging, including anatomical superimposition and suboptimal visualization conditions. While evidence shows that structured training can improve diagnostic accuracy,^5^ broad implementation is limited. Thus, the development and validation of AI-assisted diagnostic tools for panoramic images offer a promising pathway to reduce inter-reader variability and support more consistent clinical decision support.

Recent advances in deep learning architectures have enabled automated caries detection on dental radiographs, including segmentation-based approaches using U-Net variants and attention mechanisms.^6^^,^^7^ Such systems have demonstrated high accuracy ranging from 86% to 95% in controlled evaluations, yet clinical validation remains limited. Independent assessments of commercially available systems have reported variable sensitivity and only moderate inter-rater reliability,^8^ underscoring the gap between algorithmic performance and real-world clinical effectiveness.

Rigorous clinical validation – particularly through reader studies assessing impacts on dentists’ diagnostic accuracy and workflow efficiency – is still lacking. Furthermore, current evidence provides insufficient insight into whether AI assistance can reduce experience-based performance gaps or preserve high specificity in routine practice, where noncarious radiolucencies often mimic true lesions.

To bridge this translational gap, we have developed a dual-stage U-Net-based system that combines automated tooth localization with caries detection. In this multicentre diagnostic reader study, we evaluated whether AI assistance improves diagnostic performance and workflow efficiency for caries detection on panoramic radiographs. We hypothesized that assistance would (1) increase sensitivity for early-career dentists without compromising specificity; (2) shorten interpretation time; and (3) move early-career performance closer to that of experienced dentists. We further evaluated standalone system performance relative to unassisted readers to contextualize potential utility in screening workflows.

## Materials and methods

### Study design and workflow

A multicentre diagnostic reader study was conducted across three tertiary oral medical centres in China (Peking University School and Hospital of Stomatology, Tongji Medical College of Huazhong University of Science and Technology affiliated Tongji Hospital, and the Third Hospital of Hebei Medical University) from January to May 2022. The protocol was approved by the institutional ethics committee of Peking University School and Hospital of Stomatology (Approval No. PKUSSIRB-202277093a) with written informed consent obtained from all participants.

The study dataset consisted of 402 anonymized panoramic radiographs acquired under routine clinical conditions from the three participating centres. Images were obtained according to each institution’s standard acquisition protocol and were required to meet predefined image-quality criteria. The multicentre design allowed inclusion of variations in patient characteristics and image acquisition conditions while maintaining consistent eligibility criteria across centres.

The reader study protocol, endpoints, and statistical analysis plan were defined prior to evaluation.

Fifteen dentists participated in the reader study, including 12 early-career dentists (≤3 years of clinical experience) and three senior dentists (>10 years of experience). Each participant interpreted the same set of 402 anonymized panoramic radiographs under four reading conditions:

1. **unassisted** (manual interpretation only);
2. **assisted** (with on-screen system output available);
3. **system-only** (AI inference without human input); and
4. **expert reference** (consensus of three senior experts).

**Fig. 1:**
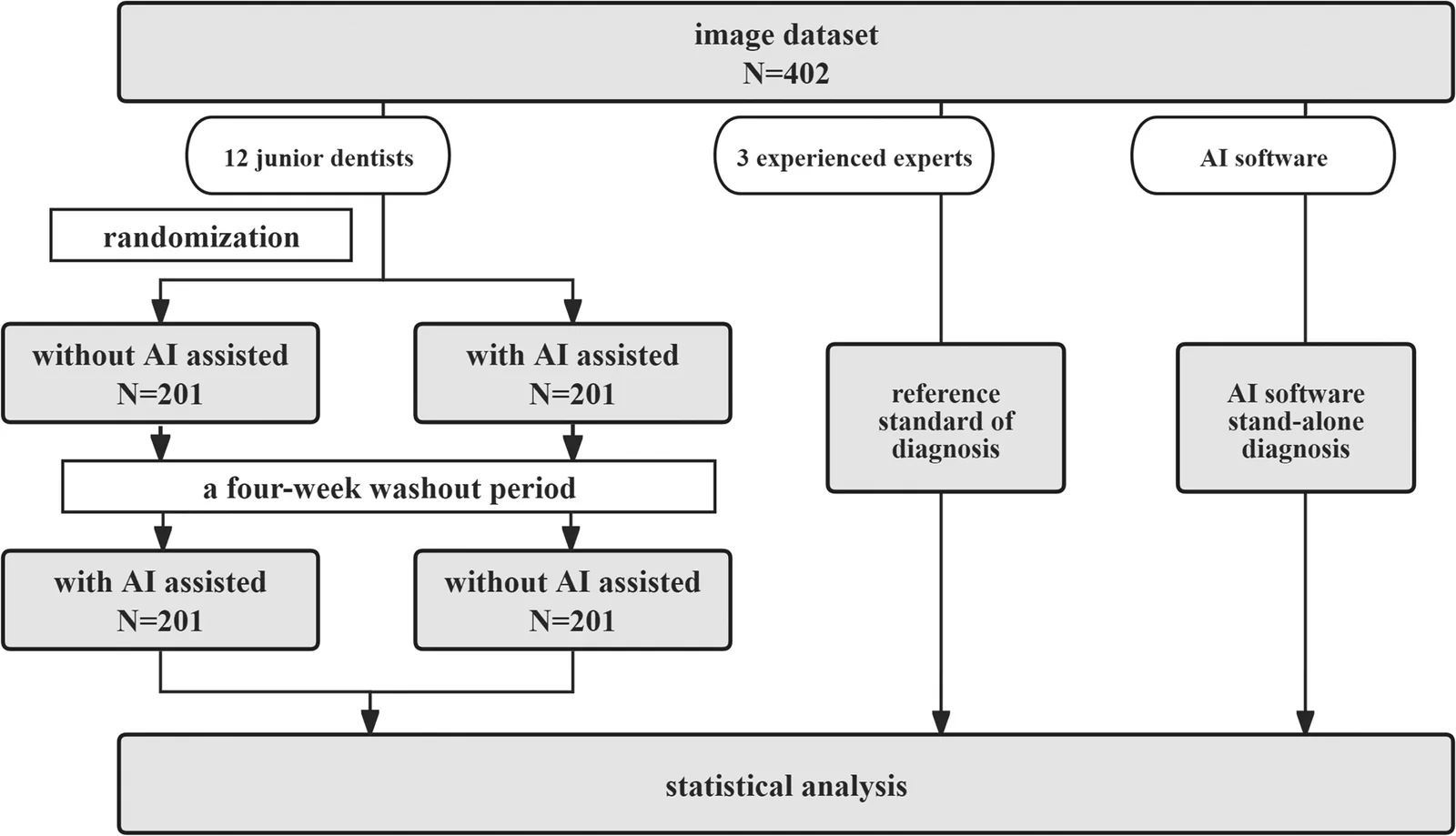
Study design and reader workflow. Each participant interpreted the same 402 panoramic radiographs under four conditions: unassisted, assisted, system-only, and expert reference. The study followed a crossover design with a 4-week washout period to minimize recall bias. Ground truth was established by three senior experts. Interpretation time was automatically recorded.

During AI-assisted interpretation, readers were allowed to review each radiograph independently before optionally activating the AI overlay. The system output was presented as colour-coded contours and candidate lesion highlights, without confidence scores or probability estimates. The overlay was superimposed on the original radiograph and could be toggled on or off, allowing sequential review of the unassisted image and the AI-assisted display. The AI-generated lesion boundaries could not be edited by the readers. Final diagnostic decisions were made solely by the readers. The order of the radiographs was independently randomized for each reading session. Readers were blinded to their previous annotations and diagnostic decisions, which were not displayed or otherwise accessible during the subsequent session. Two reading sessions were conducted, separated by a 4-week washout period to minimize recall bias. Readers were randomized to one of two interpretation sequences: unassisted followed by AI-assisted reading, or AI-assisted followed by unassisted reading. Reading time was automatically logged from image opening to case submission. The study flowchart is shown in Figure 1. All participants received detailed guidelines for image interpretation and annotation (Supplementary Material).

### Image acquisition and inclusion criteria

Panoramic radiographs were obtained using five standardized devices: Carestream Dental CS 8000C (Carestream Dental), KaVo OP 2D (KaVo), NewTom VGi (Quantitative Radiology), Planmeca ProMax (Planmeca), and Sirona Orthophos XG (Dentsply Sirona). All images were deidentified to remove personal and institutional identifiers and stored in DICOM, JPEG, PNG, BMP, or TIFF formats. Inclusion criteria required patients ≥18 years old with diagnostic-quality images: full mandibular visibility without motion artefacts (signal-to-noise ratio ≥25 dB), resolution ≥800 × 1200 pixels (8-bit greyscale), and average greyscale values 70 to 180. Exclusion criteria included incomplete dental arches, full-crown restorations (>50% crown coverage), residual roots only, and images previously used for AI model training.

### AI system development

**Fig. 2:**
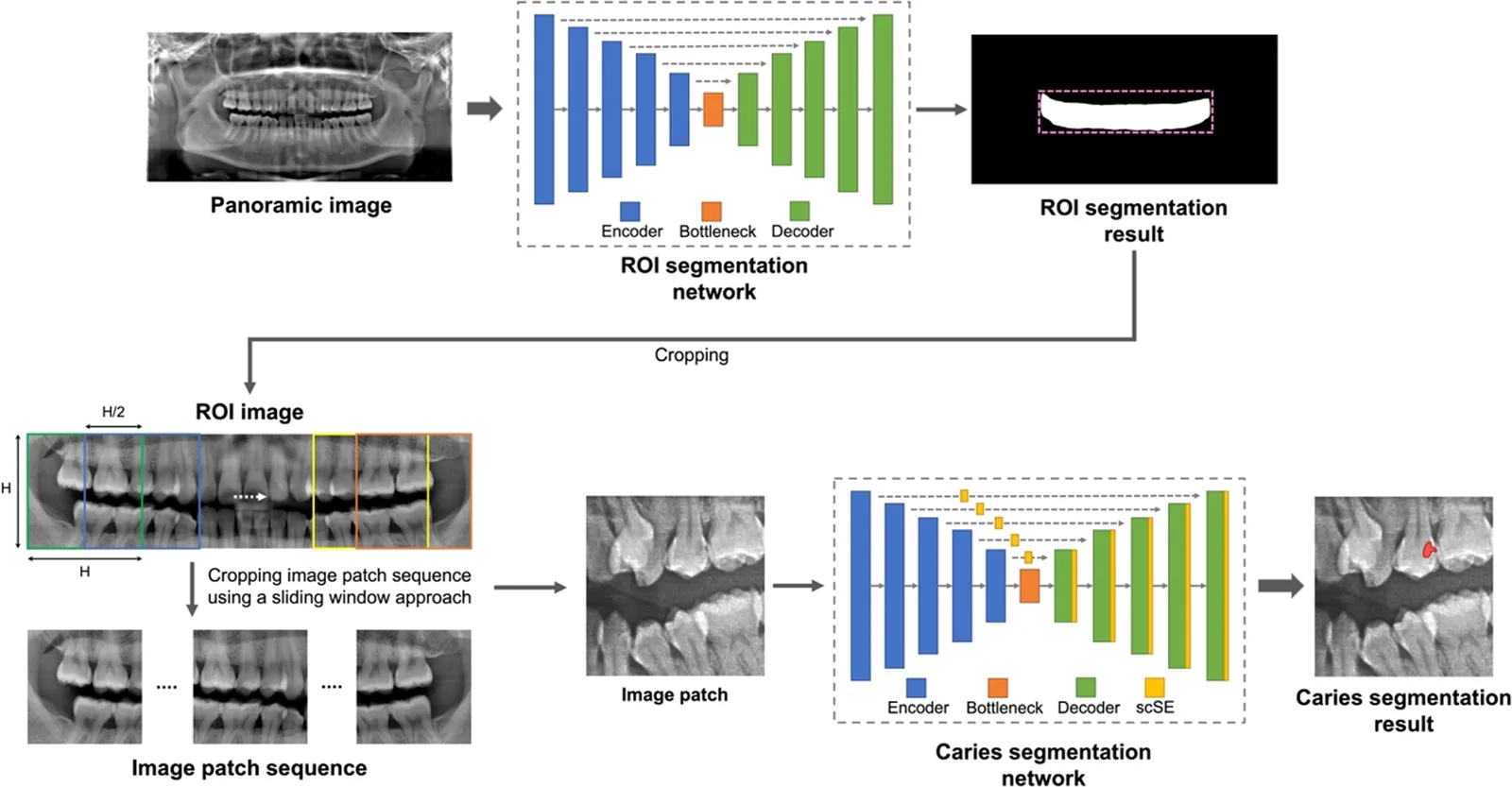
Architecture of the dual-stage U-Net model for AI-assisted caries detection. The first stage segments the dento-alveolar region of interest (ROI) from the full panoramic image. The second stage performs caries segmentation within ROI patches using an scSE-enhanced decoder.

The AI assistance system was based on a dual-stage U-Net framework, illustrated in Figure 2. In the first stage, a segmentation network localized the dentoalveolar region of interest,^9^ followed by a second network for caries detection within cropped image patches. An attention-enhanced decoder was incorporated to improve feature weighting.^10^

The model was trained on 4617 labelled panoramic radiographs that were randomly divided into training, validation, and internal test sets (70%, 10%, and 20%, respectively). To address class imbalance, composite loss functions combining Dice and cross-entropy components were applied.^11^^,^^12^ Model performance was optimized using standard deep learning procedures and evaluated on the internal test set prior to clinical reader study implementation.

All implementation was performed in PyTorch.^13^ All model development and validation were completed before the multicentre clinical evaluation to avoid data leakage between training and reader study datasets.

### Reference standard and lesion definition

The expert reference standard was established by three experienced dentists with over 10 years of clinical experience. Before formal annotation, the experts participated in a calibration session in which the diagnostic criteria, lesion definitions, and annotation procedures were jointly reviewed and discussed to ensure consistent interpretation. Each expert then independently performed pixel-wise annotations using a dedicated in-house tool under standardized conditions,^14^ while blinded to the other experts’ assessments, without additional diagnostic aids or clinical records. Interexpert agreement was quantified using Fleiss’ kappa based on the independent annotations. Final labels were determined by a two-of-three majority vote. Cases with discordant annotations or unresolved differences were subsequently reviewed in a consensus meeting until agreement was reached.

Carious lesions were identified as radiolucent areas extending beyond the dentino-enamel junction and stratified by depth following the American Dental Association Caries Classification System^15^: Small: limited to the DEJ or outer third of dentin; Moderate: extending to the middle third; Advanced: reaching the inner third or approaching the pulp.

### Statistical analysis

An a priori sample size calculation was performed using the modified Obuchowski–Rockette model and parameter estimates from a pilot study. With 12 readers, at least 223 caries-positive cases were required to detect superiority in tooth-level sensitivity, assuming a 10% between-condition difference, a one-sided *α* of 0.025, and 85% power. For tooth-level specificity, at least 96 caries-negative cases were required for a noninferiority analysis with a prespecified margin of −10%, a one-sided *α* of 0.025, and 95% power. Allowing for screening misclassification and possible exclusions, 402 cases were included.

Tooth-level sensitivity and specificity were analysed using a modified Obuchowski–Rockette multireader, multicase model, with reading condition treated as a fixed effect and reader-related effects treated as random effects. The covariance matrix was estimated using 10,000 bootstrap resamples. Reading time was analysed within the same multireader framework. A hierarchical testing strategy was prespecified to control the family-wise type I error rate: the key secondary endpoint of reading time was formally tested only after both primary endpoints – superiority in sensitivity and noninferiority in specificity – were met. Other secondary outcomes were considered supportive analyses. The −10% noninferiority margin was prespecified to ensure that improved sensitivity would not be accompanied by an excessive loss of specificity, while accounting for variability observed in the pilot study.

### Data and code availability

Due to patient privacy restrictions, the original radiographs are not publicly available. Anonymized data may be shared upon reasonable request to the corresponding author, subject to institutional and ethical approval.

## Results

### Study population

A total of 402 panoramic radiographs from adult patients were included (187 males, 215 females; mean age = 39.5 ± 14.8 years). The study cohort included patients with a broad age distribution and varying lesion severity, and images were acquired using multiple panoramic imaging systems across the three participating institutions.

Of the 402 panoramic radiographs, 229 (57.0%) were classified as caries-positive and 173 (43.0%) as caries-free. At the tooth level, 447 of 11,172 teeth (4.0%) were classified as carious (Table 1).

**Table 1:** Comparative performance metrics of caries detection at the tooth level and case level.

Metric	Tooth level			Case level		
Sample size	11,172 teeth			402 radiographs		
	Unaided	AI-assisted	Difference (95% CI)	Unaided	AI-assisted	Difference (95% CI)
Sensitivity	67.4%	82.4%	+15.0%* (95% CI, 9.8-20.2)	84.7%	93.3%	+8.6%* (95% CI, 2.0-15.1)
Specificity	97.2%	97.4%	+0.2% (95% CI, −0.3 to 0.7)	61.0%	59.8%	−1.2% (95% CI, −9.2 to 6.8)
PPV	42.4%	47.9%	+5.5%* (95% CI, 1.7-9.4)	57.3%	58.7%	+1.4% (95% CI, −3.3 to 6.1)
NPV	99.1%	99.5%	+0.4%* (95% CI, 0.2-0.6)	88.0%	93.9%	+5.9%* (95% CI, 1.6-10.1)
AUC	0.825	0.901	+0.076* (95% CI, 0.038-0.114)	0.770	0.819	+0.049* (95% CI, 0.005-0.093)
Time (s)	-			65.1	50.4	−14.7* (95% CI, −24.2 to −5.3)

AUC, area under the receiver operating characteristic curve; NPV, negative predictive value; PPV, positive predictive value.

⁎ *P* < .05.

### Reference standard reliability

Inter-expert agreement for the reference standard based on the three independent annotations was excellent (Fleiss *κ* = 0.82; 95% CI, 0.79-0.85). Among early-career dentists, mean pairwise agreement improved from *κ* = 0.61 (95% CI, 0.57-0.65) without assistance to *κ* = 0.73 (95% CI, 0.69-0.77) with assistance, reflecting improved diagnostic reproducibility.

### Tooth-level performance

AI assistance increased sensitivity by 15.0% (82.4% vs 67.4%; Δ = 15.0%, 95% CI 9.8-20.2%, *P* < .001) while maintaining specificity (97.4% vs 97.2%; Δ = 0.2%, *P* = .379).

Positive predictive value (PPV) increased from 42.4% to 47.9% (Δ = 5.5%, 95% CI 1.7%-9.4%, *P* = .005), suggesting that teeth identified as carious were more likely to be truly carious when AI was utilized. Negative predictive value (NPV) also increased from 99.1% to 99.5% (Δ = 0.4%, 95% CI 0.2%-0.6%, *P* < .001), enhancing confidence in identifying noncarious teeth.

The area under the receiver operating characteristic curve (AUC) also increased from 0.825 to 0.901 with AI assistance (Δ = 7.6%, 95% CI, 0.038-0.114, *P* < .001), indicating an overall improvement in diagnostic accuracy.

The standalone AI system achieved a sensitivity of 79.2% (95% CI, 76.0-82.4), specificity of 98.4% (95% CI, 98.1-98.7) at the tooth level, demonstrating strong diagnostic performance as an adjunct tool.

Stratified analysis revealed consistent improvements across caries severities: sensitivity increased by 21.1% for moderate caries (58.0%→79.1%, *P* < .001) and 14.1% for advanced caries (68.7%→82.8%, *P* = .002) (Table 2).

**Table 2:** Stratified analysis by caries severity.

Severity	Sensitivity difference (% points)	95% CI	*P*
Moderate	+21.1	11.4-30.8	<.001
Advanced	+14.1	7.4-20.8	.002

Moderate and advanced caries were defined based on radiographic depth following the American Dental Association Caries Classification System.

### Case-level performance

At the patient level, assistance shortened mean interpretation time by 22.7% (50.4 vs 65.1 seconds; Δ = −14.7 seconds, 95% CI, −24.2 to −5.3, *P* = .003) while improving sensitivity (93.3% vs 84.7%; Δ = 8.6%, *P* = .013).

Case-level specificity did not significantly differ between assisted and unassisted conditions (59.8% vs 61.0%; Δ = −1.2%, *P* = .774) and negative predictive value improved from 88.0% to 93.9% (Δ = 5.9%; *P* = .008).

AUC rose from 0.770 to 0.819 (Δ = 0.049; *P* = .031).

AI assistance also reduced missed multilesion cases (≥2 carious teeth per patient), improving complete detection from 37.8% to 59.5% (*P* < .001).

The standalone AI demonstrated a sensitivity of 92.1%, specificity of 63.0%, PPV of 59.4%, and NPV of 93.2% at the case level.

### Performance in challenging cases

In 62 patients with noncarious defects such as attrition or wedge lesions, the system maintained specificity (95.8% vs 95.6%, Δ = 0.2%, *P* = .412) while improving sensitivity from 70.5% to 83.4% (Δ = 12.9%, *P* = .003) (Table 3). These findings indicate robust discrimination between true caries and visually similar conditions.

**Table 3:** Performance in noncarious defects.

Metric	Unaided	AI-assisted	Difference (95% CI)
Sensitivity	70.5%	83.4%	+12.9* (95% CI, 5.7-20.1)
Specificity	95.6%	95.8%	+0.2 (95% CI, −0.3 to 0.7)

Analysis was restricted to 62 cases with confirmed wedge-shaped defects or attrition.

⁎ *P* < .05.

### Individual reader performance

All 12 early-career dentists showed numerical sensitivity improvements with AI assistance (mean Δ = 15.0%, range 5.1%-37.4%). The overall MRMC analysis demonstrated a statistically significant improvement (*P* < .001). There was no significant change in specificity (mean Δ = 0.2%; *P* = .45).

Nine Readers exceeded standalone system’s performance when assisted (Table 4). The most pronounced improvement occurred in Dentist #4 (sensitivity 75.2% vs 37.8%; Δ = 37.4%). Even among the three dentists whose sensitivity did not exceed that of the AI system, substantial improvements were noted, with gains of 10.52%, 14.54%, and 37.36%, respectively.

**Table 4:** Comparison of sensitivity and specificity for each dentist, both with and without AI assistance.

Dentist	Sensitivity		ΔSensitivity	Specificity		ΔSpecificity
	Unaided	AI-assisted		Unaided	AI-assisted	
1	81.0%	87.3%	+6.3%	96.5%	96.8%	+0.3%
2	66.4%	77.0%	+10.5%	98.7%	98.6%	−0.1%
3	52.8%	82.8%	+30.0%	97.5%	97.4%	−0.2%
4	37.8%	75.2%	+37.4%	99.3%	98.7%	−0.6%
5	73.4%	85.7%	+12.3%	98.4%	98.5%	+0.2%
6	59.5%	83.2%	+23.7%	97.9%	97.4%	−0.6%
7	76.3%	85.2%	+8.9%	97.3%	97.8%	+0.4%
8	75.8%	84.1%	+8.3%	96.3%	97.3%	+1.0%
9	74.1%	79.2%	+5.1%	92.3%	94.1%	+1.8%
10	74.1%	85.2%	+11.2%	97.6%	97.8%	+0.2%
11	76.7%	88.1%	+11.4%	96.7%	96.3%	−0.4%
12	61.3%	75.8%	+14.5%	97.5%	98.0%	+0.6%
Average	67.4%	82.4%	+15.0%	97.2%	97.4%	+0.2%

Data reflect 12 early-career dentists’ diagnostic performance before and after AI assistance. Statistical inference was performed based on the overall MRMC analysis rather than individual reader comparisons. ΔSensitivity and ΔSpecificity represent change in metrics before and after assistance.

**Fig. 3:**
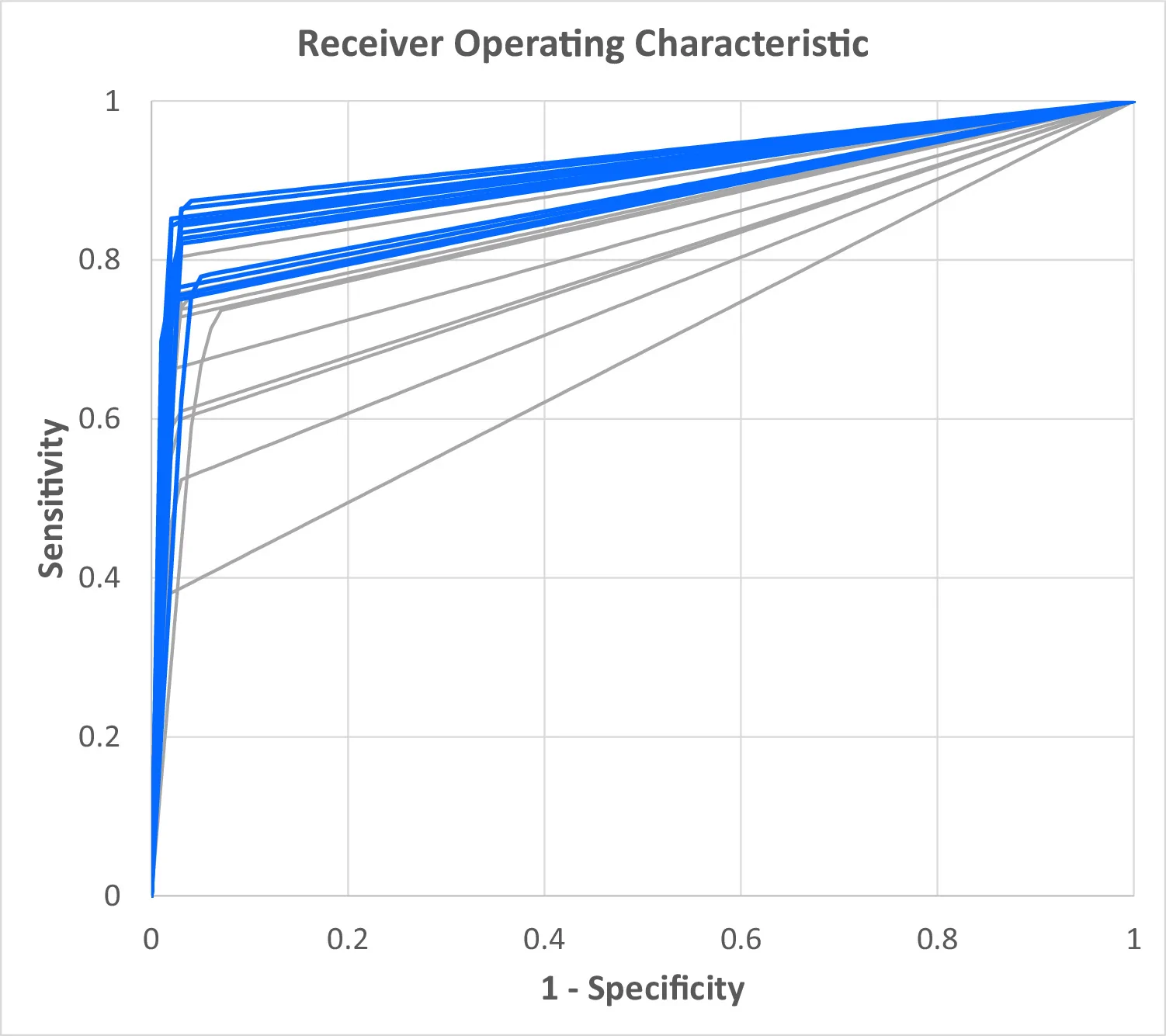
Receiver operating characteristic (ROC) curves for 12 early-career dentists, comparing unassisted (grey) and AI-assisted (blue) performance. AI assistance resulted in consistent improvement in diagnostic performance across all readers, reflected by upward AUC shifts in all curves.

Figure 3 shows receiver operating characteristic curves for each reader, illustrating consistent upward shifts after assistance and an overall AUC improvement from 0.825 to 0.901 (difference of 0.076, 95% CI, 0.038-0.114, *P* < .001).

## Discussion

Recent comprehensive reviews have highlighted both the opportunities and challenges of AI integration in dental diagnostics, emphasizing the importance of clinical validation and responsible implementation.^16^^,^^17^ This multicentre reader study demonstrates that AI assistance during panoramic radiograph interpretation improves diagnostic performance and efficiency, particularly among early-career dentists. Assisted interpretation was associated with increased sensitivity without loss of specificity, shorter reading time, and improved inter-reader agreement (*κ* = 0.61-0.73). These findings suggest that assistive systems may help narrow experience-related performance differences and support more consistent radiographic caries detection in routine dental settings.^18^^,^^19^

Our results are consistent with prior clinical evaluations of AI-supported caries assessment, which report improved lesion detection by less experienced clinicians, while also indicating that changes in specificity or decision thresholds may occur depending on the system, task definition, and clinical context.^8^^,^^18^, ^20^ Importantly, several reviews emphasize that much of the existing evidence base is still dominated by controlled or single-centre studies with heterogeneous reference standards, underscoring the need for broader validation under real-world constraints.^21^^,^^22^ In response to these limitations, the present study assessed a dual-stage framework across three tertiary centres using heterogeneous panoramic systems, which may better capture device- and setting-related variability relevant to routine practice.^16^ Comparative evidence on panoramic radiographs has also shown that AI can match or exceed less experienced clinicians on selected diagnostic tasks, with measurable time savings, although performance varies by pathology and reference standard.^23^

By improving diagnostic consistency and sensitivity in this study setting, AI assistance may reduce experience-related variability in panoramic radiograph interpretation, potentially by reshaping visual search and attention allocation during image review.^24^ Future work should move beyond diagnostic accuracy and evaluate how AI-assisted reads influence treatment thresholds and downstream clinical decision-making in routine workflows, including the risk of over- or undertreatment.^8^^,^^25^ While sensitivity improved, the modest gains in positive predictive value and the relatively low case-level specificity warrant cautious clinical interpretation. The AI system is intended only to highlight teeth and regions with suspected carious lesions on panoramic radiographs and does not provide a definitive diagnosis or treatment recommendation. In routine practice, AI-generated prompts should trigger careful clinician reassessment and should be interpreted together with clinical examination, visual inspection, and, when indicated, adjunctive imaging such as bitewing radiographs. This approach is particularly important in screening workflows, where suspicious findings should be confirmed before referral or treatment, thereby preserving the benefit of improved sensitivity while reducing unnecessary interventions arising from false-positive prompts.

A key safety-related finding is that high specificity was maintained even in the presence of noncaries defects such as attrition and wedge lesions, where radiographic artefacts and optical effects can contribute to false-positive caries calls.^26^ Sensitivity gains were consistent across moderate and advanced lesions, supporting stage-robust performance; this is clinically relevant because management decisions often depend on lesion depth assessment.^27^ Together, these results support consistent performance in the studied settings, while broader claims of clinical reliability should be confirmed through prospective and externally validated evaluations.

Oral diseases remain among the most common health conditions worldwide and continue to impose a major burden.^28^ In China, the combination of high untreated caries burden and high-throughput clinical environments underscores the need for interpretation approaches that are both consistent and time-efficient. National survey findings indicate substantial caries prevalence among adults and increasing disease burden with age.^2^ In this context, AI assistance may help reduce inter-reader inconsistency related to differing levels of experience and support more consistent decision-making across clinical environments. Operationally, the observed reduction in interpretation time suggests potential workflow efficiency gains; however, the net benefit will depend on end-to-end integration costs and human–AI interaction patterns in real-world use, which should be quantified in future studies.^24^

Strengths of this study include its multicentre design, standardized expert reference, and direct evaluation of clinical readers. Several limitations should be acknowledged. Variations in image quality and exposure may influence detection outcomes in clinical radiographs. Panoramic imaging is primarily a screening modality and does not replace intraoral or bitewing radiographs for early lesion detection and depth assessment, where higher spatial resolution is required.^27^ Assistance may mitigate – but cannot eliminate – these modality-related constraints. The order of the radiographs was independently randomized between sessions, and readers were blinded to their previous annotations. Nevertheless, despite the 4-week washout period, residual recognition or cognitive learning effects cannot be completely excluded. Exposure to AI-generated lesion outlines may have altered readers’ visual search strategies or diagnostic thresholds, and such effects could have carried over to subsequent interpretation. Although the present study included three independent tertiary centres, all participating institutions were located within China. The current datasets may therefore not fully capture variations in disease prevalence, patient demographics, imaging protocols, or scanner characteristics encountered in other healthcare settings. Although the scanner models used in the study were recorded, detailed case-level distributions by scanner model and acquisition parameters were not systematically retained in the analytical dataset. Such variations may influence AI performance when deployed across different populations or imaging environments. Future studies should include external validation using geographically and ethnically diverse cohorts and a broader range of imaging platforms to further evaluate model performance across diverse clinical environments. The number of senior dentists included as a comparator group was limited, which may restrict the robustness of comparisons across experience levels. While diagnostic accuracy was assessed, the downstream clinical impact on treatment decisions and patient-relevant outcomes requires further investigation.^8^^,^^25^ The reference standard was derived from independent expert interpretation and subsequent consensus based on panoramic radiographs alone, without clinical examination, bitewing radiography, cone-beam computed tomography, or histopathological confirmation. Although this approach is commonly used in retrospective imaging studies, reliance on the same imaging modality for both lesion assessment and reference labelling may introduce incorporation-related bias and misclassification, particularly for early carious lesions. Therefore, the expert consensus should be regarded as a radiographic proxy reference standard rather than a definitive diagnostic gold standard.^27^

Future work should validate AI-assisted interpretation stability across devices and settings, and incorporate systematic evaluation of reader–AI interaction patterns (eg, gaze behaviour and error typology) to clarify mechanisms of benefit across experience levels.^21^^,^^28^ Such efforts would support more robust and safer integration of AI tools into routine panoramic radiograph interpretation.^22^^,^^29^

Beyond diagnostic performance, successful clinical translation of AI-assisted radiographic interpretation will depend on appropriate integration into clinical workflows. Clinician acceptance and adequate training are essential to ensure that users understand AI outputs and recognize their limitations. Clear responsibility frameworks and interoperability with existing dental information systems, including picture archiving and communication systems and clinical software platforms, will also be required for safe deployment. Future implementation studies should evaluate not only diagnostic accuracy, but also usability, workflow efficiency, user experience, and long-term safety monitoring in routine clinical environments.

Ethically, the system should be framed as decision support rather than replacement of professional clinical judgment, with dentists retaining responsibility for diagnosis and treatment decisions.^30^ Real-time deployment requires compliance with applicable data governance and regulatory standards. Appropriate training is necessary to mitigate over-reliance and ensure safe clinical integration.

## Conclusion

This multicentre reader study demonstrates that AI assistance during panoramic radiograph interpretation can significantly improve the diagnostic sensitivity, efficiency, and reproducibility of early-career dentists without compromising specificity. The system’s consistent performance across lesion severities and challenging noncarious cases supports its potential role as a decision-support tool to reduce experience-related variability in routine panoramic radiograph interpretation. Broader implementation will require prospective validation across diverse imaging platforms and settings, and evaluation of its impact on downstream clinical decision-making.

## Funding

This work was supported by the Beijing Natural Science Foundation Haidian Original Innovation Joint Fund (Grant No. L242060) and the Beijing Municipal Science and Technology Project (Grant No. Z251100006025008).

## Ethics statement and informed consent

The study was approved by the Ethics Committee of Peking University School and Hospital of Stomatology (Approval No. PKUSSIRB-202277093a). Written informed consent was obtained from dentist participants, and the use of retrospective patient radiographs was approved with a waiver of individual patient consent due to minimal risk. Informed consent was obtained from all individual participants included in the study.

## Study registration

The study was conducted in accordance with Good Clinical Practice guidelines as part of a regulated clinical evaluation. Owing to regulatory requirements, the protocol was not publicly registered. Data management and statistical analyses were performed independently.

## Author contributions

*Contributed to data curation, formal analysis, and writing of the original draft*: Wu. *Contributed to data curation and project administration*: Hou. *Contributed to methodology development and software implementation*: Ding. *Contributed to formal analysis, methodology, and software development*: Xu ZN, Bai. *Contributed to project administration and validation*: Liu. *Contributed to study conceptualization, supervision, and critical review and editing of the manuscript*: Chen, Xu MM. *Contributed to study conceptualization and critical review and editing of the manuscript*: Deng. *Reviewed and approved the final manuscript*: All authors.

## Conflict of interest

The authors declare that they have no conflicts of interest relevant to this study.

## Appendix Supplementary materials

Image, application 1

## Footnotes

Supplementary material associated with this article can be found in the online version at doi:10.1016/j.identj.2026.109812.

## References

[1] World Health Organization (2022). https://www.who.int/publications/i/item/9789240061484.

[2] Wang X. (2018).

[3] Tamam N., Almuqrin A.H., Mansour S. (2021). Occupational and patients effective radiation doses in dental imaging. Appl Radiat Isot.

[4] Wenzel A. (2021). Radiographic modalities for diagnosis of caries in a historical perspective: from film to machine-intelligence supported systems. Dentomaxillofac Radiol.

[5] Pakbaznejad E.E., Pakkala T., Haukka J., Siukosaari P. (2018). Low reproducibility between oral radiologists and general dentists with regards to radiographic diagnosis of caries. Acta Odontol Scand.

[6] Alharbi S.S., AlRugaibah A.A., Alhasson H.F., Khan RU. (2023). Detection of cavities from dental panoramic X-ray images using nested U-Net models. Appl Sci.

[7] Zhu H., Cao Z., Lian L., Ye G., Gao H., Wu J. (2022). CariesNet: a deep learning approach for segmentation of multi-stage caries lesion from oral panoramic X-ray image. Neural Comput Appl.

[8] Mertens S., Krois J., Cantu A.G., Arsiwala L.T., Schwendicke F. (2021). Artificial intelligence for caries detection: randomized trial. J Dent.

[9] Xu M., Wu Y., Xu Z., Ding P., Bai H., Deng X. (2023). Robust automated teeth identification from dental radiographs using deep learning. J Dent.

[10] Roy A.G., Navab N., Wachinger C. (2018).

[11] Sudre C.H., Li W., Vercauteren T., Ourselin S., Cardoso M.J. (2017). Deep Learning in Medical Image Analysis and Multimodal Learning for Clinical Decision Support.

[12] Taghanaki S.A., Zheng Y., Zhou S.K. (2019). Combo loss: handling input and output imbalance in multi-organ segmentation. Comput Med Imaging Graph.

[13] Paszke A., Lerer A., Killeen T. (2019).

[14] Cantu A.G., Gehrung S., Krois J. (2020). Detecting caries lesions of different radiographic extension on bitewings using deep learning. J Dent.

[15] Young D.A., Nový B.B., Zeller G.G., Hale R., Tran C. (2015). The American Dental Association Caries Classification System for clinical practice: a report of the American Dental Association Council on Scientific Affairs. J Am Dent Assoc.

[16] Samaranayake L., Tuygunov N., Schwendicke F. (2025). The transformative role of artificial intelligence in dentistry: a comprehensive overview. Part 1: Fundamentals of AI, and its contemporary applications in dentistry. Int Dent J.

[17] Tuygunov N., Samaranayake L., Khurshid Z. (2025). The transformative role of artificial intelligence in dentistry: a comprehensive overview Part 2: The promise and perils, and the International Dental Federation Communique. Int Dent J.

[18] Hegde S., Gao J., Vasa R., Cox S. (2023). Factors affecting interpretation of dental radiographs: a systematic review. Dentomaxillofac Radiol.

[19] Schwendicke F., Golla T., Dreher M., Krois J. (2020). Artificial intelligence in dentistry: chances and challenges. J Dent Res.

[20] Devlin H., Williams T., Graham J., Ashley M. (2021). The ADEPT study: a comparative study of dentists’ ability to detect enamel-only proximal caries in bitewing radiographs with and without the use of AssistDent artificial intelligence software. Br Dent J.

[21] Hung M., Yevseyevich D., Khazana M., Schwartz C., Lipsky M.S. (2025). Charting new territory: AI applications in dental caries detection from panoramic imaging. Dent J.

[22] Arzani S., Karimi A., Iranmanesh P. (2025). Examining the diagnostic accuracy of artificial intelligence for detecting dental caries across a range of imaging modalities: an umbrella review with meta-analysis. PLoS One.

[23] Gunec H.G., Urkmez E.S., Danaci A., Dilmac E., Onay H.H., Cesur Aydin K. (2023). Comparison of artificial intelligence vs. junior dentists’ diagnostic performance based on caries and periapical infection detection on panoramic images. Quant Imaging Med Surg.

[24] Arsiwala-Scheppach L.T., Castner N.J., Rohrer C. (2024). Impact of artificial intelligence on dentists’ gaze during caries detection: a randomized controlled trial. J Dent.

[25] Pul U., Tichy A., Pitchika V., Schwendicke F. (2025). Impact of artificial intelligence assistance on diagnosing periapical radiolucencies: a randomized controlled trial. J Dent.

[26] Dioguardi M., Guerra C., Sovereto D. (2026). Radiographic artifacts in the diagnosis of dental caries: systematic review with meta-analysis. Oral Radiol.

[27] Kühnisch J., Aps J K M, Splieth C. (2024). ORCA-EFCD consensus report on clinical recommendation for caries diagnosis. Paper I: Caries lesion detection and depth assessment. Clin Oral Investig.

[28] Peres M.A., Macpherson L.M.D., Weyant R.J. (2019). Oral diseases: a global public health challenge. Lancet.

[29] Liu X., Rivera S.C., Moher D., Calvert M.J., Denniston A.K., DECIDE-AI Steering Group (2022). Reporting guidelines for clinical studies evaluating artificial intelligence–based decision support systems: the DECIDE-AI guideline. BMJ.

[30] Liu X., Cruz Rivera S., Moher D., Calvert M.J., Denniston A.K., SPIRIT-AI and CONSORT-AI Working Group (2020). Reporting guidelines for clinical trial reports for interventions involving artificial intelligence: the CONSORT-AI extension. Nat Med.

