# Supplementary material for: Multicentre Evaluation of AI-Assisted Caries Detection on Panoramic Radiographs Among Early-Career Dentists
Source: Int Dent J. 2026 Sep 8;76(6):109812. doi: 10.1016/j.identj.2026.109812 (PMC13579388; doi:10.1016/j.identj.2026.109812)
Supplement: Supplementary file 1 [file mmc1.docx]

Guidelines on image reading and annotation

1. Image Reading Environment

Reference Standard Dentist: For optimal image interpretation, turn off the lights and close the curtains in the image reading area to create a dim environment.

Participating Dentists: To simulate real clinical scenarios, image reading can be conducted in typical working environments, such as clinics or offices.

2. Control of Image Reading Time

Simulate real clinical conditions by adhering to the dentists’ usual image reading habits, avoiding any deliberate changes due to study participation.

Participating dentists must record the time taken to interpret each image (from opening the image to completing the interpretation). To maintain accuracy, avoid interruptions during the reading process, such as needing to temporarily close the current image.

3. Scope of Image Evaluation

The statistical analysis will include only natural permanent teeth.

Exclude the following: deciduous teeth, supernumerary teeth, teeth with restorations, residual roots, residual crowns (defects reaching 2/3 or more), and teeth that have undergone endodontic treatment.

All permanent teeth are counted as 32. The reference standard dentist will identify and record unrepaired missing teeth, implants, prostheses, endodontically treated teeth, residual roots, and residual crowns, which will be excluded from the analysis to determine valid teeth for statistical evaluation. Teeth with overlapping areas greater than one-third, making them unidentifiable, will not be interpreted.

The reference standard dentist will annotate carious lesions on the valid teeth included in the statistical analysis.

After training, participating dentists can annotate caries on the teeth, excluding the aforementioned five categories. If they incorrectly annotate teeth belonging to these categories, such teeth will be classified into the respective categories according to the reference standard and excluded from the statistical analysis.

4. Definition of Dental Caries

Dental caries, also known as tooth decay, is a progressive lesion of the hard tissues of the teeth caused by the combined action of multiple factors in the oral cavity. It is characterized by the demineralization of inorganic substances and the decomposition of organic substances, evolving from color changes to the formation of significant lesions as the disease progresses.

Radiographic Features of Dental Caries: Areas of decreased density in the crown or root, with blurred boundaries and possibly irregular shapes.

5. Situations Excluded from Caries Interpretation

1) Artifacts generated during imaging.
2) Tooth Wear:

Manifested as low-density areas with clear edges on the occlusal surfaces of the crowns, appearing flat or as pits.

Wedge-shaped Defects: Located at the cervical portion of the tooth, manifested as low-density areas with sharp, clear edges, resembling an acute triangle.

3) Dental Hard Tissue Defects: Low-density images with irregular crown morphology and clear edges.
